# Piperacillin-Induced Immune Hemolysis Presenting with Tachycardia and Cardiac Arrest

**DOI:** 10.1155/2011/816497

**Published:** 2011-12-22

**Authors:** Ghan-Shyam Lohiya, Lilia Tan-Figueroa, Vamsi Krishna

**Affiliations:** ^1^Department of Public Health, Fairview Developmental Center, Costa Mesa, CA 92626, USA; ^2^Department of Cardiology, Cedars-Sinai Medical Center, Los Angeles, CA 90048, USA

## Abstract

A 20-year-old nonverbal patient with profound developmental disabilities was treated with intravenous piperacillin-tazobactam for respiratory infection. After 8 days, he became afebrile with normal pulmonary status, *but his pulse remained inexplicably rapid (114/minute)*. Investigations revealed severe normochromic normocytic hemolytic anemia (hemoglobin: 40 g/L, reticulocytes: 9.4%, nucleated erythrocytes: 5%). While being hospitalized, patient experienced sudden cardiac arrest from which he was successfully resuscitated. He had no blood loss or intrinsic heart disease to explain the acute anemia or cardiac arrest. He had uneventfully received piperacillin-tazobactam on 7 occasions during the preceding 5 years for >50 days. Patient was treated with intravenous crystalloids, methylprednisolone and transfusion of 3 units of packed erythrocytes. Piperacillin-tazobactam was discontinued. A direct antiglobulin test was positive for immunoglobulin G and complement. Antibody to piperacillin was detected in patient's serum by the “immune-complex” method confirming “piperacillin-induced immune hemolytic anemia (PIHA)”. On discharge (day 15), patient's hemoglobin improved to 115 g/L (baseline: 131 g/L). Vigilant *clinical* and hematological monitoring for anemia is indicated in piperacillin-treated patients, particularly in those unable to verbalize their discomfort. Repeated piperacillin exposure may sensitize and predispose patients to PIHA.

## 1. Introduction

Pulmonary and urinary infections are common causes of morbidity and mortality. Piperacillin-tazobactam (Zosyn), a synthetic penicillin-beta lactamase combination, is widely used to treat these infections. Rarely, such treatment may become complicated by the development of piperacillin-induced immune hemolytic anemia (PIHA) [1–10]. Whereas most afflicted patients help their physicians suspect PIHA by reporting anemia symptoms, some not due to severe disability, serious illness, or medicine side effects. Resultant delay in treatment may be fatal. We report such an unusual presentation of PIHA in a nonverbal patient where the only clue to its diagnosis was an inexplicable tachycardia, later complicated by cardiac arrest.

## 2. Case Presentation

Our patient lived in a facility for ~400 people with severe developmental disabilities. He was 20 years old, bedridden, and unable to speak. He had a permanent tracheostomy and feeding gastrostomy. He experienced frequent pulmonary infections requiring intravenous antibiotics. Daily he received 10 mg montelukast and ipratropium-albuterol inhalations for bronchospasm, 10 mg hydrocortisone and 75 mcg levothyroxine for panhypopituitarism, 30 mg levetiracetam and 90 mg phenobarbital for tonic clonic epilepsy, and 10 mg baclofen for spastic quadriplegia. His baseline hemoglobin was 131 g/L and core body temperature subnormal (36°C).

On day 1 of this illness, patient developed fever (38.3°C or 101°F), respiratory congestion, and leukocytosis. He was treated with 3 g piperacillin-375 mg tazobactam (Zosyn 3.375 g) intravenously every 6 hours. His pulmonary condition gradually improved. On day 9, he had no fever, but his pulse remained inexplicably rapid at 114 (usual: 80) per minute. Testing revealed severe anemia (hemoglobin: 40 g/L, hematocrit: 0.10, reticulocytes: 9.4%, nucleated erythrocytes: 5%, hypochromasia, spherocytosis; Table 1). While being hospitalized for this acute anemia, patient became pulseless and apneic. He was successfully resuscitated from this sudden cardiac arrest. Patient's subsequent treatment included intravenous crystalloids and methylprednisolone, transfusion of three units of packed erythrocytes, and discontinuation of piperacillin-tazobactam.

Investigations on day 9 revealed the following anomalies: serum lactate dehydrogenase 412 (normal 88–230) u/L, serum bilirubin 24 (normal <21) *μ*mol/L, erythrocyte sedimentation rate 140 (normal 1–10) mm/h, arterial blood carboxyhemoglobin 3.1 (normal: <9% of total hemoglobin, or in this case 0.36) g/dL, presence of warm antibodies in serum, and positive direct antiglobulin test for both immunoglobulin G (IgG) and complement. Serum tests were negative for immunoglobulin M antibody against cytomegalovirus, parvovirus and mycoplasma. Hemoglobinuria was absent indicating that hemolysis was not intravascular. *Serum collected on day 15 was positive for antibody to piperacillin by the “immune-complex” method*, in which patient's serum was tested with erythrocytes in the presence of a solution of piperacillin. Tracheal culture grew alkaligenes xylosoxidans (multiple-antibiotic-resistant gram-negative bacilli) sensitive to and uneventfully treated with tobramycin. Tracheal culture was negative for influenza A and B, parainfluenza 1, 2, and 3, and adenovirus. On discharge (day 15), patient's hemoglobin was 115 g/L. 

This patient had uneventfully received piperacillin-tazobactam on 7 occasions during the preceding 5 years for a cumulative total of >50 days. He had no history of heart disease, blood loss, hemolytic anemia, blood transfusion, Raynaud's disease, hepatosplenomegaly, systemic lupus erythematosus, paroxysmal cold or nocturnal hemoglobinuria, or of treatment with another hemolysis-associated drug. 

## 3. Discussion 

Was this patient's severe anemia caused by piperacillin? We used the Naranjo algorithm to address this critical issue [11]. The specific scores on the ten Naranjo questions were: Existence of previous conclusive reports of PIHA (score: 1), occurrence of anemia after piperacillin administration (score: 2), improvement upon piperacillin discontinuation (score: 1), no alternative cause for anemia (score: 2), no recurrence of anemia with placebo (Score: 1), and objective confirmation of anemia (score: 1); answers to questions 4, 7, 8, and 9: no or not done (score: 0). The final tally of 8 qualified this patient's anemia as a “probable” adverse drug reaction to piperacillin. Piperacillin will not be administered to this patient in the future. 

Development of new anemia in patients treated with multiple medicines should automatically arouse a suspicion of an iatrogenic etiology. Of the ~125 drugs inducing hemolysis, the commoner ones are cefotetan, ceftriaxone, ticarcillin-clavulanate, ampicillin-sulbactam, levodopa, methyldopa, and quinidine [12–16]. A handy clue to hemolysis in nonsmokers is an elevated arterial blood carboxyhemoglobin, a test commonly performed on critically ill patients [12]. Carbon monoxide is endogenously generated in equimolar quantities during heme degradation, and its production increases commensurate with hemolysis. 

This patient had a positive direct antiglobulin test indicating the presence of IgG autoantibodies and/or complement bound to the erythrocyte surface, and the diagnosis of autoimmune hemolysis. With the knowledge of this patient's history, the probable cause of such hemolysis had to be a drug, and piperacillin was the most likely culprit among the active medicines in this case. Detection of piperacillin-antibody was confirmatory; however, such testing is available only in specialized centers [13]. 

“Warm antibodies” in the patient's serum on day 9 probably were not autoantibodies because the patient never had “idiopathic warm type autoimmune hemolytic anemia” either before or after the current piperacillin episode. Those warm antibodies were probably piperacillin (drug-dependent) antibodies that were reacting because of the in vivo presence of piperacillin in the patient's blood on day 9 [10, 12–16]. Although a test for warm antibodies was not performed in this case after discontinuation of piperacillin, such test would probably have been negative due to subsequent loss of piperacillin. Since warm antibodies hemolyze erythrocytes at body temperature (≥37°C), the anemia in PIHA is usually severe, as in this patient. Piperacillin, like penicillin, acts as a hapten to form stable piperacillin-erythrocyte membrane complexes. Complement activation amplifies the immune reaction. 

Piperacillin has been marketed since 1993, with $797 million in sales in 2010. PIHA was not identified during piperacillin's clinical trials, but has been documented during its postmarketing experience [17]. Precise incidence of PIHA is not known, but PIHA is probably rare as only about 40 cases (including two deaths) have been described in the literature [1–10]. Nearly one-third of the PIHA cases, especially the severe ones have occurred in patients with cystic fibrosis, a population prone to recurrent pulmonary infections just like tracheostomy patients. The latency between start of piperacillin treatment and diagnosis of PIHA has commonly been 7–13 days. Both genders have been affected equally, and the patients' ages have ranged between 23 and 68 years [1–10]. 

There are two striking similarities between our patient and several published cases: *prior piperacillin treatment on multiple occasions* and *latency* between piperacillin initiation and development of anemia [1–10]. *We hypothesize that repeated piperacillin treatment may predispose to PIHA by providing multiple opportunities for hapten-antigen challenge and the activation of immune hemolysis. Perhaps repeated piperacillin courses should be avoided, and piperacillin-treated patients should be monitored with frequent blood counts.*

Our patient had no prior heart disease, yet he experienced sudden cardiac arrest—a near-fatal event. This was obviously precipitated by the anemia-related severe myocardial hypoxemia in a heart whose oxygen demand had increased due to tachycardia. We credit our patient's survival to our facility's heightened emphasis on clinical monitoring–*an astute employee's attention to patient's tachycardia* was the only clue to something being awry on day 9. 

Our patient experienced a massive 70% reduction of his hemoglobin within 8 days! Most patients with such serious illness would facilitate their diagnosis by reporting anemia-related symptoms (asthenia, palpitation, dyspnea, chest discomfort, or malaise). Our patient could not do so due to his severe disability which in his case was preexisting! However, even many previously healthy patients may also become unable to “partner” with their physicians due to mental obtundation from serious illness or medicines. Thus, this case also highlights the need for extreme vigilance while caring for individuals with severe disabilities or* serious illnesses*. 

## Figures and Tables

**Table 1:** Hematological parameters in the patient with severe piperacillin-induced immune hemolytic anemia.

Day	Hematocrit %	Hemoglobin g/L	Erythrocyte ×10^12^/L	Leukocyte ×10^9^/L	Reticulocytes %
1	39.2	131	4.34	13.6	Normal
9	10.4	40	1.32	19.5	9.4
10	24.8	91	2.92	19	7.4
14	35.6	115	3.65	7.1	Normal
327	38.6	132	4.2	6.6	—
Normal	39–49	136–175	4.7–6.1	4.8–10.8	0.5–1.5
